# Randomized clinical trial of the *individualized coordination and empowerment for care partners of persons with dementia* (ICECaP) intervention: impact on preparedness for caregiving

**DOI:** 10.1007/s40520-025-02959-z

**Published:** 2025-03-01

**Authors:** Virginia T. Gallagher, Shannon E Reilly, Anna Arp, Agustina Rossetti, Ryan Thompson, Carol A. Manning

**Affiliations:** 1 Department of Neurology, University of Virginia, PO BOX 801018, Charlottesville, VA 22908 USA; 2 Brain Institute, University of Virginia, Charlottesville, VA USA

**Keywords:** Caregiver, Care partner, Behavioral intervention, Preparedness

## Abstract

**Background:**

Dementia care partners are at elevated risk of adverse mental health outcomes and often feel unprepared for their caregiving role. Individualized Coordination and Empowerment for Care Partners of Persons with Dementia (ICECaP) is an intervention that involves one-on-one individualized support from a dementia care coordinator for a dementia care partner. At least once monthly contact is made from a dementia care coordinator to the dementia care partner by telephone, video conferencing, email, and/or in-person support.

**Aims:**

We aimed to determine whether ICECaP improves care partner readiness and whether improvements in readiness are associated with mental health improvements.

**Methods:**

In this randomized control trial of ICECaP, *n* = 61 care partners completed 12-months of the ICECaP intervention, and *n* = 69 care partners received routine clinical support (controls) in an outpatient memory care clinic (ClinicalTrials.gov: NCT04495686). We compared changes in care partner preparedness, dementia knowledge, and self-efficacy from baseline to 12-months between ICECaP and controls.

**Results:**

ICECaP care partners improved on self-reported preparedness for caregiving from baseline to 12-months to a significantly greater degree versus controls (*p* =.001, η_p_^2^ = 0.066); no group differences were detected on change in dementia knowledge or self-efficacy over time. Exploratory analyses revealed that within the ICECaP group, longitudinal improvement in preparedness was significantly associated with longitudinal decreases in self-reported caregiving burden and negative reactions to behavioral symptoms of dementia (corrected *p*s < 0.05).

**Discussion/Conclusions:**

ICECaP significantly improves dementia caregiver preparedness, which is associated with improved mental health.

**Supplementary Information:**

The online version contains supplementary material available at 10.1007/s40520-025-02959-z.

## Introduction

Over 11 million family members and friends provide unpaid care for older adults with dementia in the United States. Currently, family/friend dementia caregiving is valued at $350 billion and 18.4 billion hours of care per year nationally [1]. Although many dementia care partners experience meaning and fulfillment in the context of their caregiving role, caring for an older adult with dementia is associated with increased levels of psychosocial stress, depression, and emotional burden [2–9]. Among dementia caregivers, poorer mental health is associated with lower levels of caregiving preparedness, confidence, and dementia knowledge [10–12].

Many programs and interventions have been developed to support dementia care partners and improve their mental health, quality of life, and caregiving readiness. Among them, collaborative care coordination has emerged as a promising, individualized intervention to help care partners and their care recipients with dementia navigate complex health systems, financial/insurance systems, community resources, and advanced care planning. Importantly, care coordinators also provide care partners with social and emotional support [13–16]. We developed an intervention for dementia care partners called ICECaP: Individualized Coordination and Empowerment for Care Partners of Persons with Dementia. ICECaP involves individualized elements of care coordination, supportive counseling, resource options counseling, psychoeducation, and skills training and is delivered in a hybrid setting. ICECaP involves an initial home visit (in-person optional), followed by at least monthly contacts via phone, email, HIPAA-compliant video calls, and/or in-person support visits (e.g., accompaniment to clinic visits for the older adult with dementia). A unique aspect of ICECaP relative to other care coordination interventions is that ICECaP dementia care coordinators engage in weekly group supervision with a licensed psychologist and dementia care partner science expert.

ICECaP contains important elements of the new voluntary nationwide model established by the Centers for Medicare & Medicaid Services in 2023 called the “Guiding an Improved Dementia Experience (GUIDE) Model.” Namely, programs that participate in GUIDE are required to have care navigators (akin to dementia care coordinators in ICECaP), who have specialized training in dementia, assessment, and care planning and who are not required to have advanced degrees. Additionally, dementia patients in GUIDE Model programs are required to have a care coordination plan, similar to plans and goals established in ICECaP. Thus, continued testing and refinement of ICECaP can provide evidence-based data to help inform policy and programming at the national level regarding care coordination and navigation services for people with dementia and their care partners.

In this study, we assess the impact of ICECaP on dementia caregivers’ preparedness for caregiving, dementia knowledge, and overall self-efficacy. Prior research has repeatedly identified dementia care partners’ desire and need for knowledge and information about multiple dementia-related topics (e.g., disease progression and healthcare, legal, and financial systems) [17–20] at multiple stages of dementia disease progression [21, 22]. Additionally, dementia care partners’ knowledge, preparedness, and self-efficacy has been shown to be responsive to psychoeducational interventions [10, 12, 23–25]. Further, as stated above, preparedness, knowledge, and self-efficacy are associated with caregivers’ levels of mental health distress and burden.

In this pilot, randomized control trial (RCT) of ICECaP, we test whether 12-months of ICECaP improves care partner preparedness, dementia knowledge, and self-efficacy. Specifically, we test the hypothesis that care partners who received 12-months of ICECaP have greater improvements in self-reported dementia knowledge, preparedness for caregiving, and self-efficacy from baseline to 12-months relative to care partners in the control group. We also explore relationships among outcome measures within the ICECaP group.

## Methods

This study was approved by the University of Virginia Institutional Review Board for Health Sciences Research (HSR#190081) and was registered with ClinicalTrials.gov: NCT04495686. All participants underwent informed consent prior to the initiation of study procedures.

### Recruitment

As reported in the published protocol [26] and the feasibility and acceptability paper for the pilot ICECaP clinical trial [27], care partners were recruited from the University of Virginia’s multidisciplinary Memory and Aging Care Clinic (MACC) when accompanying a patient with dementia to a clinical appointment. Care partners were required to be aged ≥ 18 years, possess basic spoken and written English skills, have home-based internet access, and self-identify as the primary care partner for a patient diagnosed with mild to moderate dementia (of various etiologies) living in the community (e.g., not living in a continuing care facility). Care partners were randomly assigned using a random permuted block randomization scheme to 12-months of ICECaP or 12-months of routine clinical care (controls).

### Sample and attrition

As previously published [27], of the *n* = 169 care partners recruited into the RCT who completed baseline assessments, 23.08% were withdrawn from the final sample because within 12-months, the person with dementia died (*n* = 9), moved to a higher level of care (*n* = 8), or moved out of state (*n* = 1); or the care partner chose to withdraw (*n* = 4), was lost to follow-up despite two attempts to contact (*n* = 13), or did not complete 12-month assessments (*n* = 4). The final sample of care partners included *n* = 69 controls and *n* = 61 ICECaP who completed baseline and 12-month assessments. See S1 Consort Flow Diagram.

### Procedures and intervention

Please see protocol paper [26] for details regarding study design and intervention protocol. Questionnaires listed in Table 1 were completed by both ICECaP and control group care partners at baseline and 12-months after baseline. Measures were collected online and stored using REDCap [28], hosted by the University of Virginia. Data were monitored by the clinical research coordinator for completion.

**Table 1:** ICECaP Randomized Clinical Trial measures among Dementia Care partners (CPs)

	Construct	Self-report Measure	Measure Details
*Outcome Measures*: Evaluated Baseline vs. 12-month, ICECaP vs. Control			
Readiness	CP Caregiving Preparedness	Preparedness for Caregiving Scale (PCS) [29]	8-item measure that assesses CPs’ feelings of preparedness for multiple domains of caregiving; there are five item response options ranging from 0 (“Not at all prepared”) to 4 (“Very well prepared”); total scores and average scores are reported.
	CP Dementia Knowledge	Dementia Knowledge Assessment Tool Version 2 (DKAT2) [30]	21-item measure assessing CPs’ foundational-level knowledge of dementia (e.g., dementia progression, support, and care)
	CP Self-Efficacy	General Self-Efficacy Scale (GSES) [31]	10-item measure assessing feelings of self-efficacy
Mental Health and Quality of Life Measures	CP Burden	Zarit Burden Interview (ZBI)-Short Form [32]	12-item measure assessing degree of CP burden
	CP Depression	Center for Epidemiologic Studies Depression Scale—Revised (CESD-R) [33]	20-item measure assessing symptoms of depression
	CP Anxiety	Geriatric Anxiety Inventory (GAI) [34]	20-item measure assessing symptoms of anxiety
	CP Reaction to Behavioral Symptoms	Revised Memory and Behavior Problems Checklist (RMBPC) [35]	24-item measure assessing CP-reported problematic behaviors in PWDs and CPs’ reaction to these behaviors
	CP Quality of Life	WHO (Five) Well-Being Index (WHO-5)* [36]	5-item measure assessing dimensions of psychological well-being
Covariates	CP-reported PWD Basic ADLs	Katz Index of Independence in Activities of Daily Living (Katz) [37]	6-item measures assessing independence in basic self-care daily activities
	CP-reported PWD Complex ADLs	Lawton Instrumental Activities of Daily Living Scale (Lawton) [38]	8-item measure assessing independence in complex daily activities

Note: All measures were completed by care partners in the ICECaP and control groups at baseline and 12-months.

*Intervention.* After baseline questionnaires were completed, for those in the ICECaP group, a trained dementia care coordinator (referred to going forward as care coordinator) contacted the care partner to schedule the initial phone, video, or in-person contact. Care coordinators contacted the care partner at least once per month via email, telehealth phone/video, and/or an in-person meeting for at least 15 min per session. During these sessions, care coordinators provided supportive counseling, information, resources, and related services (e.g., behavioral management, safety strategies, case management, healthcare referrals, psychoeducation) based on the individualized needs of the care partner. Care partners were encouraged to contact care coordinators as needed (i.e., no monthly limit on contacts). After the initial session, care coordinators were also required to attend regular follow-up appointments in the Memory and Aging Care Clinic with the care partner and their associated care recipient with dementia. These follow-up appointments typically occurred every 6 to 12 months.

*Control group.* They completed the same questionnaires at baseline and 12-months after baseline as the ICECaP group.

All care partners included in both the intervention and control groups in this study received follow-up care in the UVA Memory and Aging Care Clinic (MACC). Follow-up care in MACC typically involves a one-hour appointment for the patient with dementia and their care partner(s) one to two times per year with a multidisciplinary team of physicians, neuropsychologists, neuropsychology postdoctoral fellows, a nurse practitioner, a pharmacist, an occupational therapist, and a speech-language pathologist, among other specialties. While these appointments are scheduled for the person with dementia, the care partner who accompanies the patient receives information about behavioral management strategies, long-term care planning support, dementia psychoeducation, psychotherapy, support groups, adult day care, continuing care, respite facilities, and other local resources. Further, care partners are often provided emotional support, validation, and encouragement.

### Statistical analysis

Baseline differences in demographic and caregiving characteristics between ICECaP and control groups were examined to determine the need for sensitivity analyses on main efficacy tests. Baseline group comparisons were conducted via the Welch two-sample t-test, Wilcoxon two-sample Rank Sum test, and Pearson two-sample exact test, as indicated.

The hypotheses and analytical plan for the study analyses presented here slightly deviate from the original plan put forth in the protocol paper for the pilot ICECaP RCT [26]. This is due to the null findings yielded from mental health outcomes (manuscripts detailing mental health outcomes results are currently in-press). Here, we evaluate whether ICECaP improves care partner readiness by comparing change scores from baseline to 12-months between ICECaP versus controls on self-reported measures of caregiving preparedness, dementia knowledge, and self-efficacy using linear mixed ANCOVA models, after accounting for baseline differences in Katz and Lawton measures of functional independence. If change scores were significantly different between groups on any of the identified outcome measures (e.g., caregiving preparedness, dementia knowledge, and self-efficacy), we explored correlations between change on the specified outcome measure and change on other outcome measures within the ICECaP group – caregiving burden and care partner depression, anxiety, reaction to behavioral symptoms of dementia, and quality of life – using Spearman’s Rho correlations. Spearman’s Rho was selected due to the non-linear distribution of the variables. Significance testing was adjusted using the False Discover Rate (FDR) Benjamini-Hochberg procedure [39].

## Results

### Baseline results

There were no significant group differences on baseline demographic and caregiving characteristics between the ICECaP and control groups (ps > 0.05 across comparisons; see Table 2).

**Table 2:** Care Partner demographic and caregiving characteristics

	ICECaP (*n* = 61)	Controls (*n* = 69)	
Years of age, M(SD)	64.8 (12.3)	64.9 (11.6)	
Gender (women)	42 (68.9%)	52 (75.4%)	
Race/Ethnicity			
Non-Hispanic White	57 (93.4%)	60 (87.0%)	
Non-Hispanic Black/African American*	3 (4.9%)	4 (5.8%)	
Hispanic/Latino*	1 (1.6%)	3 (4.3%)	
Non-Hispanic American Indian*	0 (0.0%)	1 (1.4%)	
Highest level of education completed			
High school diploma or GED	5 (8.2%)	5 (7.2%)	
Some college*	10 (16.4%)	8 (11.6%)	
Associate’s degree*	4 (6.6%)	8 (11.6%)	
Bachelor’s degree^	27 (44.3%)	25 (36.2%)	
Master’s degree^	11 (18.0%)	15 (21.7%)	
Doctoral degree^	4 (6.6%)	6 (8.7%)	
Employment status			
Retired*	24 (39.3%)	39 (56.5%)	
Full-time^	15 (24.6%)	15 (21.7%)	
Part-time^	9 (14.8%)	2 (2.9%)	
Unemployed	2 (3.3%)	3 (4.3%)	
Monthly income			
$1–$4,999	24 (39.3%)	19 (27.5%)	
$5,000–$9,999	13 (21.3%)	18 (26.1%)	
$10,000–$14,999	4 (6.6%)	5 (7.2%)	
$15,000–$24,999	4 (6.6%)	11 (15.9%)	
Relationship to PWD			
Spouse/Partner	44 (72.1%)	49 (71.0%)	
Adult Child	14 (23.0%)	16 (23.2%)	
Co-dwelling with PWD	47 (77.0%)	54 (78.3%)	
CP hours/month supporting basic ADLs for PWD, M(SD)	27.3 (54.7)	41.8 (74.6)	
CP hours/month supporting complex ADLs for PWD, M(SD)	59.0 (68.2)	83.8 (88.9)	
Katz Index of Independence in (Basic) ADLs for PWD, M(SD)	5.6 (0.9)	5.3 (1.1)	
Lawton-Brody Instrumental (Complex) ADL Scale for PWD, M(SD)	3.9 (2.0)	3.6 (1.8)	

Note. Data presented as *n* (% of group) unless otherwise noted. There were no significant group differences on any variable listed. ADLs = activities of daily living; CP = care partner; M(SD) = Mean (Standard Deviation); PWD = person with dementia. Subgroups denoted by * and ^ were grouped together, respectively, for adequate sample size to compare demographic and sample characteristics between ICECaP versus Controls.

There were no significant differences in baseline scores on any outcome measures between groups (see Table 3). Within both groups at baseline, average preparedness for caregiving scores suggested an average response of “Not too well prepared” (1) to “Somewhat well prepared” (2) on each of the eight questions about preparedness [Mean (SD): ICECaP 1.86 (0.82); Controls: 1.92 (0.68)]; *total* preparedness scores are presented in Table 3. Baseline preparedness for caregiving scores within this sample were within the range of previously published means among CPs of older adults with dementia and other disabling conditions [40–42]. Baseline mean self-efficacy scores within each group fell in the moderate range [i.e., General Self-Efficacy Scale (GSES) total score 30–32 out of 40]. Average baseline dementia knowledge scores suggested a mean of 61% accuracy on the 21-question test.

**Table 3:** ICECaP intervention 12-Month Efficacy results

	ICECaP (*n* = 61)			Controls (*n* = 69)			
**Construct**	**Baseline** Mean (SD)	**12-Months** Mean (SD)	Mean ∆ [95% CI]	**Baseline** Mean (SD)	**12-Months** Mean (SD)	Mean ∆ [95% CI]	p
Caregiving Preparedness	14.90 (6.56)	19.36 (5.74)	4.46 [3.09, 5.83]	15.35 (5.40)	16.97 (5.45)	1.62 [0.33, 2.91]	0.001
Dementia Knowledge	13.52 (3.55)	14.82 (2.93)	1.25 [-0.56, 3.06]	13.79 (3.39)	14.60 (2.52)	1.00 [-0.44, 2.44]	0.955
Self-Efficacy	32.30 (4.12)	31.89 (4.33)	-0.41 [-1.21, 0.39]	31.70 (4.71)	31.10 (4.79)	-0.59 [-1.72, 0.53]	0.448

Note. p-values represent uncorrected ANCOVA test comparing mean change from baseline to 12-months between ICECaP and control groups after adjusting for baseline levels of functional independence as measured by the Katz and Lawton questionnaires; highlighted cells represent *p* <.05 after FDR-correction. Outcome constructs were measured using the following questionnaires: Caregiving Preparedness = PCS total score; Dementia Knowledge = DKAT2 total score; Self-Efficacy = GSES total score.

### Longitudinal results

*ICECaP engagement.* To briefly review previously published findings on engagement [27], care partners in the ICECaP group had an average of two contacts per month with their respective care coordinator, averaging a total of 26.5 contacts across 12 months. Contacts between the care partner and the care coordinator within one month ranged from 0 to 15 contacts, indicating a significant amount of variability in intervention dosage across care partners.

*ICECaP efficacy.* From baseline to 12-months, caregiving preparedness scores improved significantly more among ICECaP versus controls (see Table 3 and Supplementary Fig. 1). There were no significant group differences on dementia knowledge or self-efficacy change scores. Results were consistent when Katz Index of Independence in ADLs and/or Lawton-Brody Instrumental ADL Scales were included as covariates in analyses.

*Exploratory results.* Given that there was a significant group difference in change in caregiving preparedness in main hypothesis testing above, we evaluated the extent to which change in caregiving preparedness was correlated with changes in care partner mental health and quality of life from baseline to 12-months within the ICECaP group. Improvement in caregiving preparedness was significantly associated with decreased caregiving burden, decreased negative reactions to behavioral symptoms of dementia, and care partner anxiety, from baseline to 12-months (see Table 4).

**Table 4:** Relationship between change in Caregiving Preparedness and Change on Measures of Mental Health from Baseline to 12-months among ICECaP Care partners

		Burden ∆	Depression ∆	Anxiety ∆	ReactiontoBehavioral Dementia Sx ∆	Quality of Life ∆
Caregiving Preparedness ∆	r_s_	− 0.309	-0.141	− 0.255	− 0.344	0.087
	p	0.016*	0.284	0.048	0.009*	0.510

Note. r_s_ = Spearman’s Rho correlation; p-values represent uncorrected correlational results; * indicates *p* <.05 after FDR-correction; ∆ = change in outcome measures, representing the difference scores between 12-month and baseline assessments; Caregiving Preparedness = PCS total score; Burden = ZBI SF total score; Depression = CESD-R total score; Anxiety = GAI total score; Reaction to Behavioral Dementia Sx = RMBPC total reaction score; Quality of Life = WHO-5 total score.

*Correction for multiple comparisons*: In accordance with the statistical procedures outlined in the Methods section, eight statistical tests were conducted, including evaluation of change scores from baseline to 12-months among ICECaP versus controls on (1) caregiving preparedness (2) dementia knowledge, and (3) self-efficacy; due to the significant group differences on caregiving preparedness over time, we then calculated correlations within the ICECaP group between caregiving preparedness and change in caregiver (4) burden, (5) depression, (6) anxiety, (7) reaction to behavioral symptoms of dementia, and (8) quality of life. Uncorrected p-values are reported above in Tables 3 and 4. After FDR-correction, p-values remained significant across all eight tests conducted, with the exception of the correlation between caregiving preparedness change and anxiety change (Table 3). This relationship was no longer significant after FDR-correction.

## Discussion

**Fig. 1:**
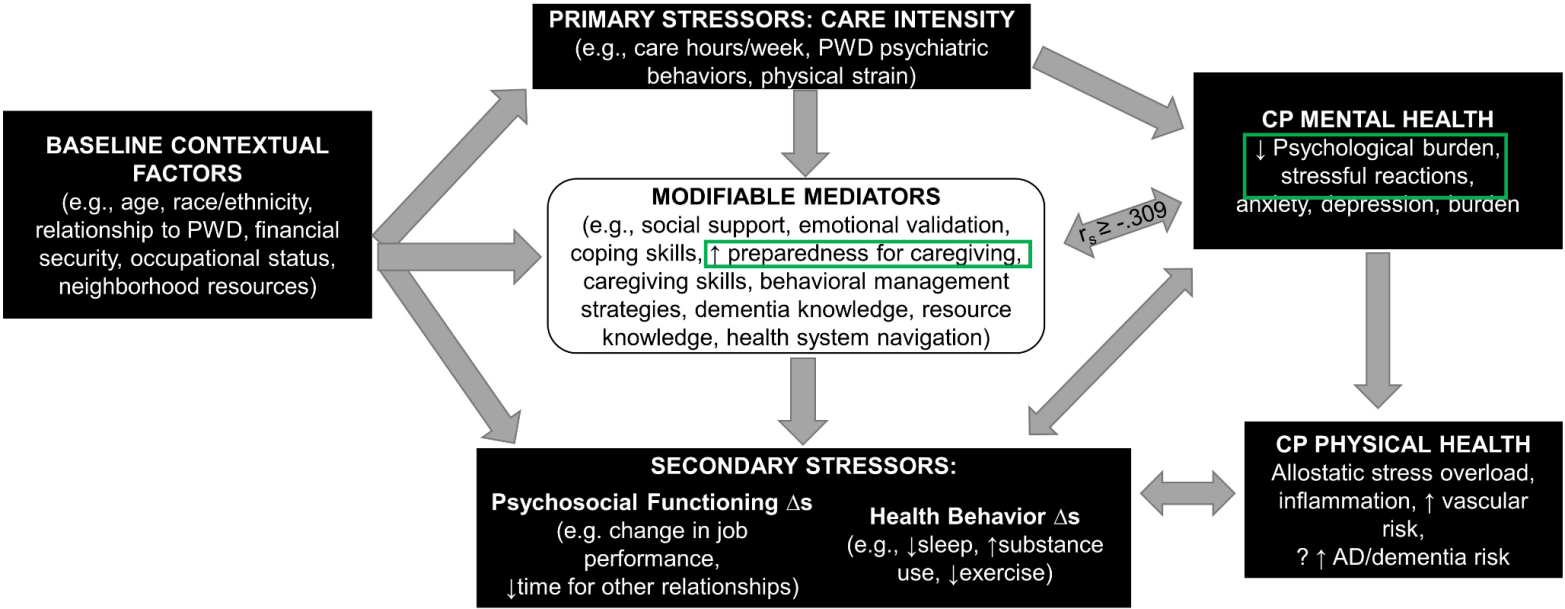
Theoretical care partner stress framework [26] with quantitative relationships from this study highlighted. *Note*. PWD = person with dementia; AD = Alzheimer’s disease; r_s_ = Spearman’s Rho correlations between preparedness for caregiving and caregiving psychological burden, stressful reactions to the behavioral symptoms of dementia, (enclosed in green boxes).

Our pilot RCT of ICECaP demonstrated that, compared to a control group, 12-months of ICECaP had a significant impact on longitudinal change in care partners’ self-reported preparedness for caregiving, but not on their dementia knowledge nor general self-efficacy. Further, care partner preparedness improvements are correlated with improved care partner mental health, specifically, lower care partner psychological burden and decreased negative reactions to the behavioral symptoms of dementia. These findings add to our theoretical care partner stress framework, originally published in the ICECaP protocol manuscript [26], by providing quantitative support to the relationship between preparedness for caregiving and aspects of care partner mental health; see Fig. 1.

Caregiving preparedness, as measured by the Preparedness for Caregiving Scale (PCS), reflects perceived readiness for multiple domains of caregiving, including providing physical care, emotional support, resource identification and utilization, and coping with the stress of caregiving. Through dementia care coordinators, the ICECaP intervention provides dementia care partners with support for social/emotional adjustment, health system navigation, and resource identification, among other components. It is not surprising that care partners in the ICECaP group indicated significant improvement in caregiving preparedness as a result of the intervention, given the focus of the dementia care coordinators on identifying resources and strategies for current issues and simultaneously planning for the future.

ICECaP care partners’ improvement in caregiving preparedness aligns with ICECaP care partners’ self-reported satisfaction with the ICECaP intervention [27]. Specifically, care partners in the ICECaP group were given a satisfaction survey about their participation in ICECaP, with response options ranging from “strongly agree” (1) to “strongly disagree” (5) [27]. Over 95% of care partners in the ICECaP group “strongly agreed[d]” that their dementia care coordinator provided them with access to community resources for the care partner and/or the care recipient. It is possible that having access and knowledge about available resources yields improvements in feeling “well prepared” to face the care-recipients’ physical, emotional, health, and functional needs and to manage the stress of caregiving. Certainly, there appear to be correlations between improved feelings of preparedness for multiple aspects of caregiving and improved psychological burden and decreased negative psychological reactions to behavioral symptoms of dementia. This is important because improved psychological burden and health are associated with improved caregiver physical health and quality of life [43, 44].

Results from this study indicate that ICECaP, which involves at least monthly contact with a dementia care coordinator, provides significant improvement in care partner preparedness for the caregiving role, above and beyond the robust standard care provided in a multidisciplinary memory and aging care clinic, which includes multidisciplinary appointments one to two times per year. This finding can help guide future study design to evaluate the efficacy and efficiency of different components of GUIDE Model programs, including multidisciplinary team care and one-on-one care coordination services. Specifically, a study investigating effective components of GUIDE Model programs might consider caregiving preparedness as a primary target of the care coordination component of the program.

ICECaP’s lack of efficacy on care partner dementia knowledge is likely multi-factorial. There have been significant advances in the science of dementia and dementia care since the DKAT2 was published over 10 years ago. As such, some of the DKAT2 items now appear outdated. For example, per the manual, the correct answer to item 8 on the DKAT2, “Knowing the likely cause of dementia can help to predict its progression,” is *No* [30]. However, we now know that different etiologies of dementia are associated with differential progression rates and survival times [45, 46]. Further, the correct answer to item 17 on the DKAT2, “When a person who has dementia is distressed, it may help to talk to them about their feelings”, is *Yes*. The utility of talking about distress or distressing stimuli with the care-recipient is extremely variable across older adults with dementia and therefore, the correct answer to this question may vary depending upon the care partner and care-recipient. Therefore, it is possible that the DKAT2 lacks validity and reliability as a measure of up-to-date dementia knowledge. Additionally, all care partners in the study had likely received significant education regarding the diagnosis and prognosis of their care recipients’ dementia and its etiology as part of standard care in the MACC, which may have increased baseline scores, thereby reducing the likelihood of detecting significant improvement on the DKAT2. To further underscore this point, care partners in this sample scored nearly 10% higher on the DKAT2 at baseline relative to care partners recruited from the community in California for a dementia care partner intervention study [47]. Further, nearly 77% of this sample had at least a college degree; of note, higher education levels (and health literacy) have been associated with higher levels of dementia knowledge in the absence of intervention [48]. Collectively, these data suggest that this is a highly educated sample with relatively high dementia knowledge at baseline, thus leaving little room for intervention effects. Future studies might consider utilizing the Dementia Knowledge Assessment Scale (DKAS) [49], which has up-to-date content and promising psychometric properties [50].

ICECaP’s lack of impact on self-efficacy, as measured by the GSES, is also likely multifaceted. Most notably, the GSES appears to measure temporally stable, trait aspects of self-efficacy that is are not specific to the caregiving situation. For example, the questionnaire assesses participants’ agreement with statements such as, “When I am confronted with a problem, I can usually find several solutions,” and “No matter what comes my way, I’m usually able to handle it ” [31]. These questions appear to probe broad views of one’s self-concept. Indeed, GSES scores tend to correlate strongly with other general constructs, such as optimistic self-beliefs and positive affect, but relatively weakly with specific self-beliefs about ability to follow-through on target behavioral goals, such as physical activity goals for example [51]. Therefore, it is not surprising that ICECaP would impact self-reported feelings of preparedness to handle difficult aspects of caregiving specifically (PCS scores) but not significantly affect care partners’ self-concept about their ability to manage problems in general (GSES scores). Future investigations into the impacts of care partner interventions on self-confidence to manage aspects of caregiving may wish to utilize the Caregiver Inventory [52] or the Revised Scale for Caregiving Self-Efficacy [53], which assess caregiving-specific self-efficacy.

The current study has several limitations that warrant consideration. One of the major limitations was lack of racial/ethnic and sociocultural diversity in our study sample, reducing the generalizability of our results to care partners in diverse cultural contexts. Intervention-specific limitations include the lack of a community-based control group, as all care partners in our study had some level of specialty dementia care. As stated, this likely translates to higher baseline preparedness and knowledge relative to care partners who are not already engaged in care at a specialty memory center. Finally, it is unclear how care recipients’ dementia etiology and disease stage may have affected the impact of ICECaP, as we did not collect this data as part of this clinical trial. Future research addressing these limitations will be needed to validate and extend our findings.

In summary, ICECaP – an intervention that involves at least monthly contact between a dementia care partner and a dementia care coordinator, who engages in weekly group supervision with a licensed psychologist/care partner science expert – improves caregiving preparedness in a sample of dementia care partners, who are predominantly from high socioeconomic backgrounds. Our study also reveals that improved caregiving preparedness is associated with improved burden and decreased emotional reactivity to behavioral symptoms of dementia. These results hold promise for the meaningful expansion of ICECaP to other sites and to care partners in the community who are not already connected to specialty dementia care and who may represent more diverse socioeconomic backgrounds. Finally, as systematic investigations of the cost and impact of the GUIDE model are underway, our study posits that caregiving preparedness and caregiving burden may be salient outcome measures to consider.

## Electronic supplementary material

Below is the link to the electronic supplementary material.

Supplementary Material 1

Supplementary Material 2

## Data Availability

We have made the 12-month outcome data freely available in a public repository: Gallagher, Virginia, 2024, “Individualized Coordination and Empowerment for CarePartners of Persons with Dementia (ICECaP): 12 month results”, 10.7910/DVN/YP4YY0, Harvard Dataverse, V1,UNF:6:GfAdGRjoO0YUOJlmpSwzTQ== [fileUNF].

## Declarations

### Data sharing Statement

Data will be made publicly available at the conclusion of the follow-up time point for the trial (please contact Carol Manning, cm4r@virginia.edu).

### AI Statement

No generative AI tools were used in preparation of this manuscript.
