# Supplementary figures and images for: Randomized clinical trial of the individualized coordination and empowerment for care partners of persons with dementia (ICECaP) intervention: impact on preparedness for caregiving
Source: Aging Clin Exp Res. 2025 Mar 1;37(1):64. doi: 10.1007/s40520-025-02959-z (PMC11870990; doi:10.1007/s40520-025-02959-z)

Supplementary Material

**Figure 1.**


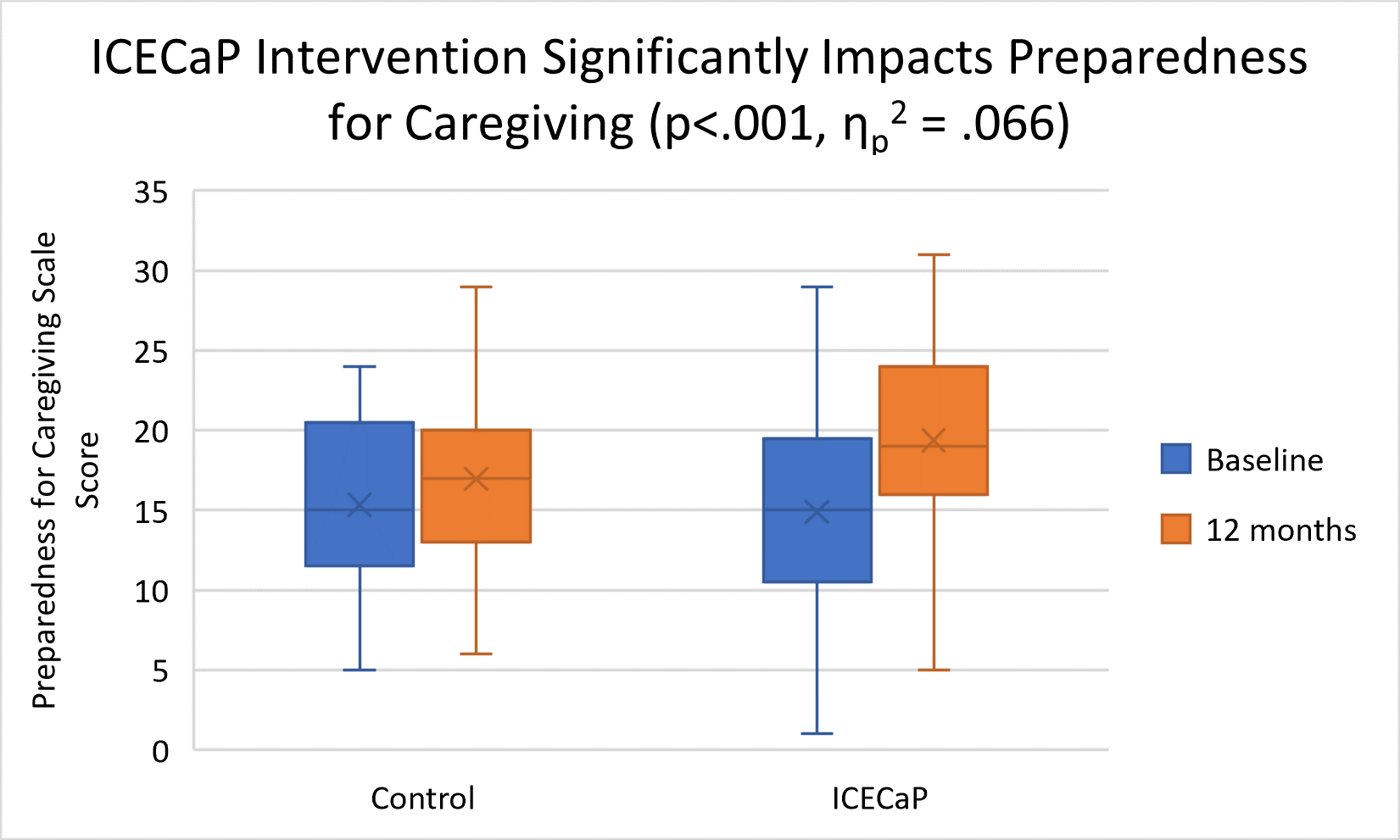

Supplement: Supplementary file 1 — Supplementary Material 1 [file 40520_2025_2959_MOESM1_ESM.docx]

## CONSORT 2010 Flow Diagram

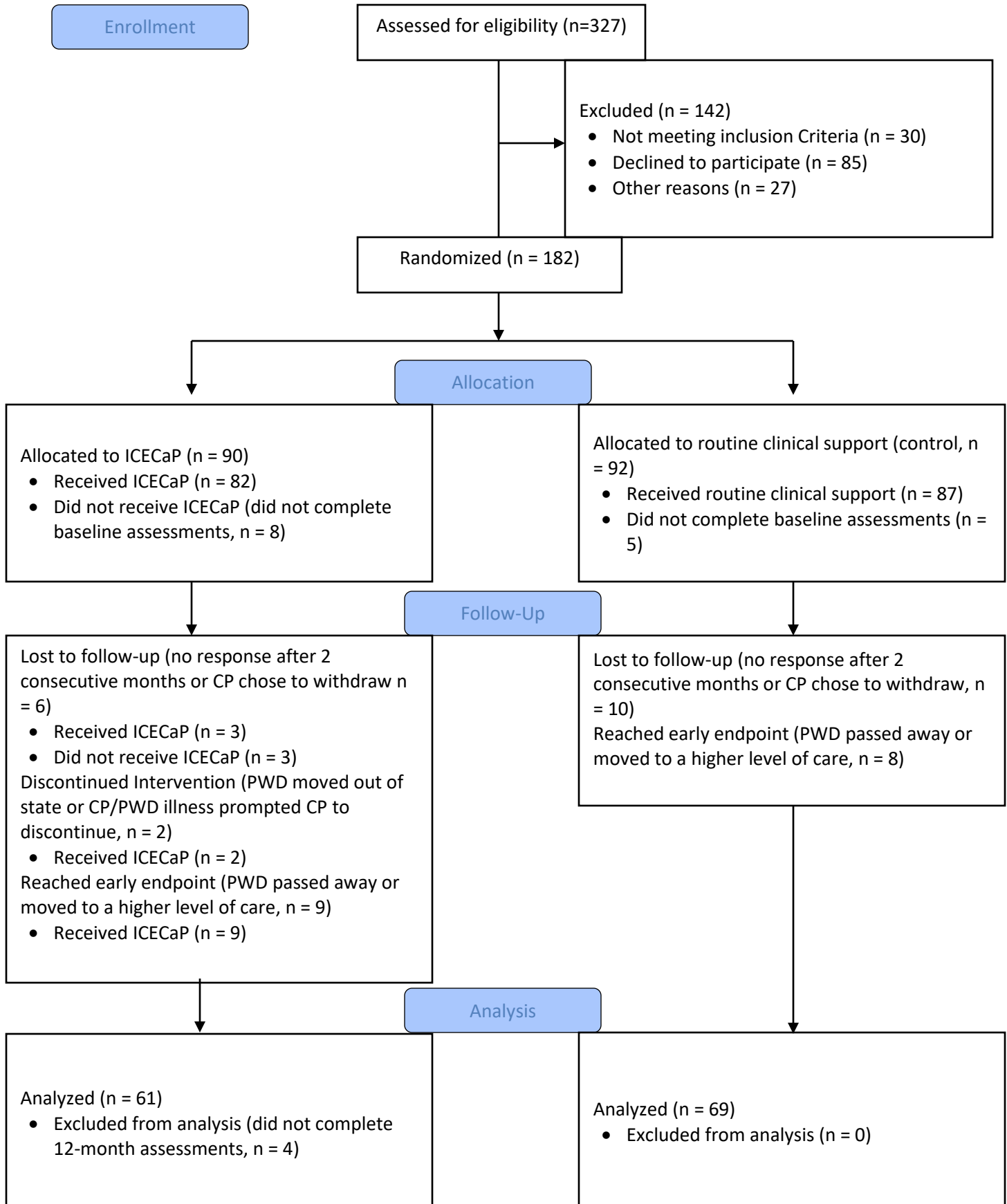

Supplement: Supplementary file 2 — Supplementary Material 2 [file 40520_2025_2959_MOESM2_ESM.pdf]
